# The Effect of Humeral Head Depressor Strengthening on Individuals with Subacromial Impingement Syndrome

**DOI:** 10.3390/medicina61112061

**Published:** 2025-11-19

**Authors:** Utku Kurtaran, Tuba Yerlikaya, Barış Yenen, Ahmet Özgül

**Affiliations:** 1 Department of Physiotherapy and Rehabilitation, Faculty of Health Sciences, Near East University, 99138 Nicosia, Cyprus; utku.kurtaran@neu.edu.tr (U.K.);; 2 Department of Physical Medicine and Rehabilitation, Kutahya City Hospital, 43100 Kutahya, Turkey; 3 Department of Physical Medicine and Rehabilitation, Kyrenia University Hospital, 99320 Kyrenia, Cyprus

**Keywords:** shoulder rehabilitation, humeral head depressors, rotator cuff, ultrasound imaging

## Abstract

*Background and Objectives*: Subacromial pain syndrome (SAPS) is one of the most common musculoskeletal problems affecting the shoulder joint. In this study, we aimed to investigate the effectiveness of a rehabilitation program targeting humeral head depressor muscles on symptoms in individuals with SAPS. *Materials and Methods*: Participants were sequentially assigned to study and control groups in a quasi-randomized design. While the control group received standard physical therapy and rehabilitation, the study group underwent a combined progressive exercise program, including humeral head depressor strengthening, peri-articular muscle exercises, scapular stabilization, and proprioceptive training. Acromio–humeral distance (AHD) and tendon thickness measurements were evaluated via ultrasonography (USG), while pain intensity, upper-extremity disability, and kinesiophobia were measured using the VAS and McGill Pain Questionnaire, DASH-T, and the Fear Avoidance Beliefs Questionnaire, respectively. *Results*: Both the study and control groups showed statistically significant increments in AHD compared to the baseline. The first and final measurements changed from 7.92 mm to 10.54 mm and from 7.72 mm to 8.41 mm, respectively. However, the increase in AHD was greater in the study group relative to the control group, and the value was statistically significant. The study group showed significant improvements in pain and disability. Kinesiophobia levels, on the other hand, decreased in both groups, but a greater decrease was observed in the study group. *Conclusions*: In this study, both the study and control groups showed an increase in AHD, but the combined exercise program targeting humeral head depressor muscles resulted in a greater improvement. Reduced tendon thickness indicated the eased motion of the rotator cuff, supporting the improvements in pain and disability. The program had a positive impact on psychosocial parameters, including pain-related kinesiophobia. Given the limited literature on the effects of such exercises on AHD and tendon thickness, this study provides an original contribution. Clinical Trial Registration: ClinicalTrials.gov identifier: NCT07228455.

## 1. Introduction

Subacromial pain syndrome (SAPS) is among the most common musculoskeletal issues [1]. SAPS usually occurs when the supraspinatus tendon is subjected to prolonged repetitive compression in the acromio-humeral space. Individuals who frequently perform overhead activities are more susceptible to this problem, which can lead to inflammation, pain, muscle and joint dysfunction that significantly impact quality of life.

The etiologic factors are intrinsic and extrinsic, including muscle weakness, acromion morphology, and scapula–humeral rhythm disturbances [2]. Physical therapy and rehabilitation modalities, along with specific exercise programs, are widely used in conservative treatment methods. Individuals who do not benefit from conservative treatment are referred to surgery [2]. In the long term, the efficacy of surgical treatments remains controversial. Therefore, selecting the appropriate patient and making an accurate differential diagnosis are crucial to maximizing the benefits of conservative approaches [3]. Individuals who experience pain during motion and joint limitation can be difficult to assess and examine [4]. To identify the factors that may cause pain, the application of differential diagnostic methods is essential.

Biomechanical abnormalities in the shoulder joint area can cause weakness of the shoulder-stabilizing structures and rotator cuff (RC) muscles [5,6,7]. As a result, the acromio-humeral distance (AHD) may decrease, and the scapulo-humeral rhythm can be disrupted. In this context, strengthening the RC muscles, which depress and stabilize the humeral head, is essential for improving shoulder biomechanics and reducing disability [5,6,7]. These muscles (mm teres major, latissimus dorsi, pectoralis major, long-head biceps brachii, supraspinatus, infraspinatus, subscapularis, and teres minor) work in coordination to maintain scapulo-humeral rhythm. Although limited, some studies show that training the humeral head depressor muscles can improve AHD [8,9]. Heron et al. compared the effectiveness of open- and closed-chain exercises with joint range-of-motion exercises, concluding that both are effective in alleviating symptoms [10]. In their electromyography (EMG) studies, Overbeek et al. demonstrated that the shoulder adductors and extensors—teres major, latissimus dorsi, and pectoral muscles—are important muscles effective in depressing the humeral head [5,6,7]. Studies investigating the effect of humeral head depressor muscle strengthening on AHD primarily focus on training the RC muscles, mainly on shoulder adductors, which have humeral-head-stabilizing and -depressing functions. We could not find any studies examining changing AHD through depressor muscle training in the literature. Thus, we aimed to investigate the efficacy of humeral head depressor training with respect to AHD and the tendon thickness of the RC.

## 2. Materials and Methods

### 2.1. Participants

This quasi-randomized controlled trial, conducted at Near East University Hospital, included individuals between the ages of 18 and 65 who presented with shoulder pain between July 2023 and July 2024 and were diagnosed with SAPS based on clinical/radiological examination. A comprehensive clinical examination, including physical and neurological evaluations, was performed by an experienced physician to determine whether there was atrophy, swelling, deformity, scapular winging, or tenderness in the shoulder joint as well as the subacromial region and acromioclavicular joint. To determine the AHD of the participants and assess the condition of the RC muscle tendons, ultrasonography (USG) was performed by an experienced specialist unaware of the participants’ clinical history. In addition, magnetic resonance images were evaluated by an experienced radiologist to rule out other disorders that cause shoulder pain. An experienced physiotherapist performed the other evaluations.

**Figure 1:**
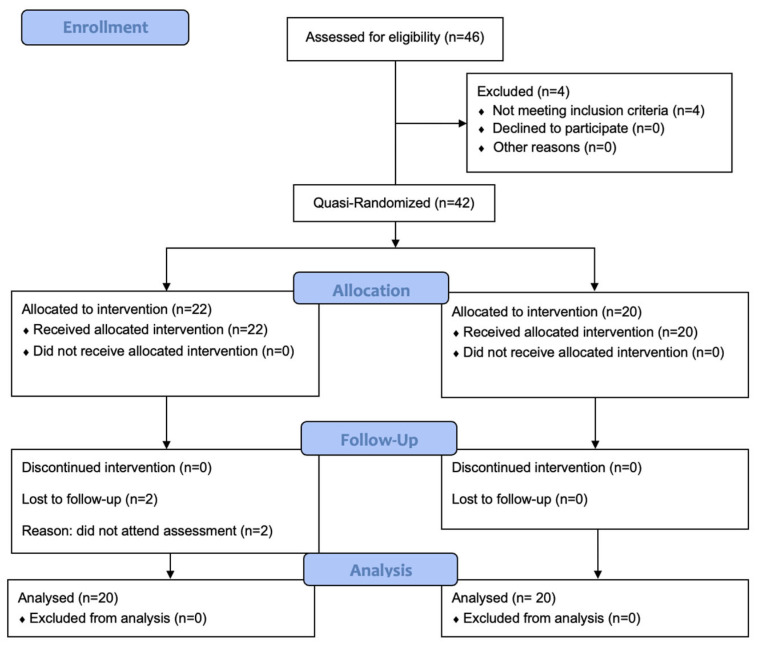
CONSORT 2025 flow diagram of the progress through the phases of this quasi-randomized trial to which the two groups were subjected (that is, enrolment, intervention allocation, follow-up, and data analysis).

Volunteers who complained of shoulder pain for more than a month; had limitations in passive movement relative to the shoulder on the other side of the body; had undergone Neer impingement, Hawkins, and Jobe supraspinatus tests; and were able to communicate were included in the study. Individuals (1) with any neurological condition affecting the upper extremities, (2) with neurological findings related to cervical disc herniation, (3) with a full-thickness rupture of one of the RC tendons, (4) with calcific tendonitis, (5) who had undergone previous shoulder surgery, (6) who had undergone physical therapy–rehabilitation for the shoulder with the same complaints in the last 6 months, or (7) who were receiving corticosteroid injections were excluded from the study. Written consent was obtained from all the participants (Figure 1).

### 2.2. Assessment Tools

The primary outcome was the change in acromiohumeral distance (AHD) measured via USG. The secondary outcomes included the thickness of the supraspinatus, infraspinatus, subscapularis, and long head of the biceps brachii tendons, along with the thickness of the general RC tendon group; pain intensity (measured via the VAS); pain characteristics (assessed via the McGill Pain Questionnaire); kinesiophobia; and shoulder function disabilities (measured using the DASH score). All outcomes were assessed before and after the intervention.

The Visual Analog Scale (VAS) and the McGill Melzack Pain Questionnaire were used to determine the participants’ pain levels. The VAS consists of a 10 cm scale, where 0 means no pain and 10 represents excruciating pain. Patients mark the line to indicate pain intensity during rest, activity, and nocturnal pain, and the results are recorded in centimeters [11]. The McGill Melzack Pain Questionnaire, on the other hand, explains pain in more detail and evaluates the sensory component, characteristics, severity, and duration of pain. In the simplest scoring system, the number of words selected in the second part of the questionnaire is between 0 and 78, and the current pain intensity in the fourth part is between 1 (mild) and −5 (unbearable). The McGill Pain Scale has been shown to be valid and reliable for the Turkish population [12].

The DASH-T questionnaire, which assesses physical impairments and symptoms, was used to assess upper-extremity disability. It is a 30-question survey that evaluates function and disability in upper-extremity injuries [13]. The first twenty-one questions assess a patient’s difficulty during activities of daily living, the next five questions assess symptoms (pain, activity-related pain, tingling, stiffness, and weakness), and the other four questions evaluate social function, work, sleep, and the patient’s self-confidence, respectively. A total score of 0–100 is obtained. High scores indicate severe disability (0 points: no disability, 100 points: maximum disability). A study of the validity and reliability of this questionnaire in Turkish has been conducted [14].

Kinesiophobia was evaluated using the Fear Avoidance Beliefs Questionnaire (FABQ). The FABQ includes 16 questions and two subscales: physical activity and work. The physical activity section is a 7-point Likert-type scale consisting of 5 questions, while the labor section consists of 11 questions, with 0 points given to the statement ‘I do not agree at all’ and 6 points given to the statement ‘I completely agree’. Both subscale scores can be used independently. The score for the physical activity section ranges from 0 to 20 points, while the range for the labor section is 0–42 points. A total score approaching 0 denotes a decrease in fear-avoidant behavior within a section, and a score approaching the maximum score indicates an increase in fear-avoidant behavior. The validity and reliability of this scale for the Turkish population have been tested [15].

USG, a portable and relatively low-cost imaging method, is based on the reflection of sound waves from tissues and does not contain ionizing radiation. It provides detailed imaging owing to the use of high-frequency (7–12 MHz) transducers for the musculoskeletal system. In recent decades, it has been widely utilized in the diagnosis of shoulder pathologies [16].

**Figure 2:**
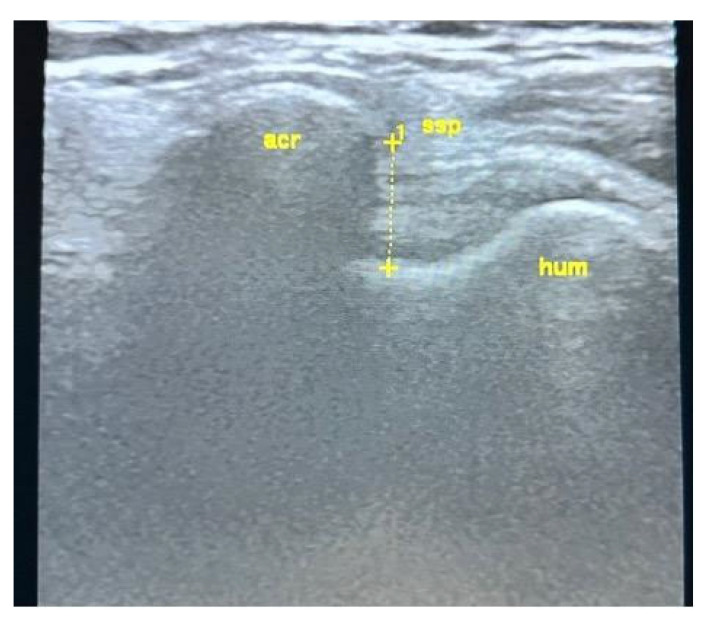
USG AHD measurements. See text.

**Figure 3:**
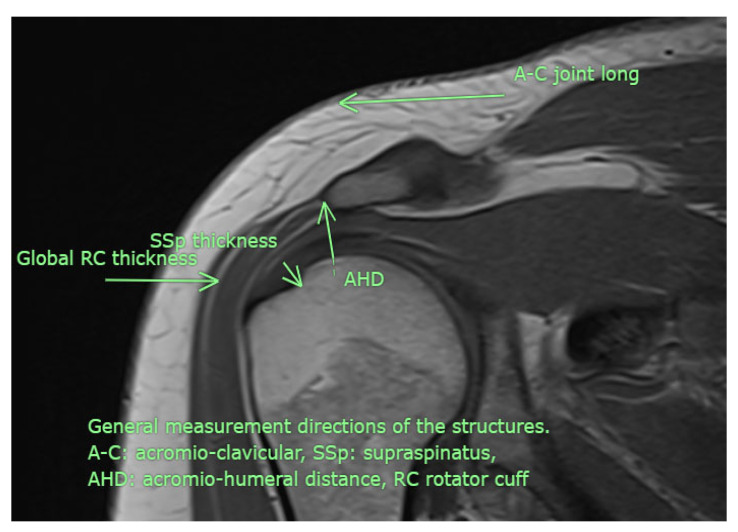
Although examined in all available directions, this MRI image illustrates the measurement sites used in the study (personal archive).

All USG evaluations were performed by a physical medicine and rehabilitation specialist with +30 years of experience in musculoskeletal USG using a GE LogiqP6 Pro device GE Ultrasound Korea, Gyeonggi, Republic of Korea and a linear transducer. Imaging was performed using standard shoulder sections while the patients were in a sitting position with an elbow flexed and an arm internally rotated and laid on the anterior thigh. We laid the probe on the coronal plane on the acromio-clavicular joint. We then moved it laterally to detect acromial-end and subacromial structures, including the subacromial bursa (SB), rotator cuff, and humeral head. Beneath the SB is the supraspinatus tendon (SSp), which attaches to the tuberculum majus of the humerus. The arm was rotated and abducted to examine the tendinous integrity and motion of the SSp. To measure the AHD and ensure standardization, a perpendicular line was drawn from the most lateral tip of the acromion to the head of the humerus, yielding an automatic value via USG-software vR2.0.5 (Figure 2 and Figure 3). In this position, we evaluated the supraspinatus and RC and measured their thicknesses on the short axis. The thickness of the SB was noted. Then, the probe was retracted, and the internal structure of the infraspinatus (Isp) and its thickness were measured. Notably, the teres minor, though difficult to discriminate, is at the posterior end of the ISp. Then, the probe was moved to the short axis to examine the biceps tendon. Here, the probe was turned to the longitudinal axis to evaluate the long biceps tendon and determine its thickness. The probe was then moved to the back side on the short axis to examine the labrum, glenoid bone, humeral head, and joint capsule. If the probe is turned longitudinally, the muscle bellies and myo-tendinous junctions of the RC muscles can be seen. Subsequently, the arm was externally rotated, and the probe was again placed medial to the biceps tendon to expose the tuberculum minus, subscapularis tendon, and anterior acromion, along with the coracoacromial ligament. Then, we positioned the arm posteriorly on the back pocket. The measurements regarding the SSp, Isp, and RC were repeated and recorded. During the examination, the acromioclavicular joint, RC tendons (supraspinatus, infraspinatus, subscapularis, and teres minor), long-head biceps tendon, SB, and related structures were evaluated in all planes. Tendon thickness, the presence of subacromial effusion, ruptures, tendinous degeneration, contour irregularities, RC integrity, and homogeneity were examined. Measurements were performed before (baseline) and after the treatment (post-tr).

### 2.3. Treatment Protocol

A total of 40 participants were included in this study. They were sequentially allocated to the intervention (*n* = 20) and control (*n* = 20) groups without any stratification or blocking constraints. Accordingly, this study is classified as a quasi-randomized controlled trial. Blinding was ensured by providing both groups with the opportunity to engage in exercise programs without disclosing the hypothesis of the study. The treatment protocol was explained by a physiotherapist with clinical experience in the field. Since the participants were allocated sequentially, the allocation sequence was not concealed. The participants were unaware of the other participants’ assignments. The physiotherapists were aware of the group allocation due to the nature of the treatment. However, the ultrasonographer performing the outcome assessments was completely blinded to group allocation, and all measurements were conducted on separate days to minimize potential bias.

The routine program was applied to both groups and consisted of strengthening, including with respect to the rotator cuff muscles. Training was performed in the direction of shoulder flexion, extension, abduction, and adduction alongside electrotherapy, ultrasound, and cold-pack applications. Different resistances and weights of the TheraBand were progressively employed as training progressed.

The study group, in addition to routine treatment, received a combined exercise program, including humeral head depressor strengthening (mm teres major, latissimus dorsi, pectoralis major, biceps brachii, supraspinatus, infraspinatus, subscapularis, and teres minor), scapular stabilization exercises, and proprioceptive training. The program was carried out progressively with various resistance weights such as the TheraBand, punching bags, and dumbbells. A scapular clock exercise carried out on the wall using a TheraBand was performed with resistance. In addition, the participants performed push-up exercises with both arms on an exercise ball placed against a wall, strengthening the RC and scapular region muscles; proprioceptive exercises were also completed. Then, an exercise consisting of pushing the wall-mounted exercise ball with one arm was also conducted, with the eyes open first and then closed. At this point, the physiotherapist pushed the exercise ball from different directions to test proprioceptive sensory awareness. All exercise sessions were performed under the direct supervision of a physiotherapist at the clinic, ensuring complete attendance in and adherence to the prescribed program. Though instructed, home-based exercises were left to the patients’ discretion; thus, compliance was fully controlled and monitored by the therapist. The starting intensity and difficulty level of each exercise were tailored to each participant based on their initial strength, range of motion, and pain tolerance. Resistance and load were then progressively increased according to each participant’s performance and ability to maintain proper technique. Both groups continued their training 3 days a week for 6 weeks, with each session lasting 45 min. The participants were evaluated before and after treatment. Post-intervention measurements were repeated at the end of the 6-week intervention period.

### 2.4. Statistical Analysis of Data

Data analysis was carried out using the Statistical Package for the Social Sciences (SPSS) 26.0.

The sample size was determined using a power analysis based on the effect size (dz = 0.67) reported in a previous study by Akkaya et al. (2017), who investigated the effects of weighted and unweighted pendulum exercises on ultrasonographic acromiohumeral distance in patients with SAPS [17]. The analysis was performed with G*Power 3.1, using a two-tailed test with 80% power and a significance level of 0.05. Accordingly, 20 participants were required in both the intervention and control groups. Considering a potential 15% dropout, we planned to have a total of 46 participants. The participants were allocated sequentially into the intervention and control groups, resulting in a quasi-randomized controlled design.

Chi-square tests were used to compare the socio-demographic characteristics and provocative clinical test measurements of the participants in the study and control groups.

The normality of the participants’ data was examined with Shapiro–Wilk tests and skewness–kurtosis values, both of which were found to exhibit a normal distribution.

An independent-samples *t*-test was used to compare the initial structural statuses of the participants in both groups.

Regarding the comparison of the baseline and final USG measurements; VAS (VAS) measurements; McGill Pain Questionnaire and Fear Avoidance Beliefs Questionnaire measurements; and Arm, Shoulder, and Hand Problems Questionnaire (DASH) measurements, a matched-sample *t* test was applied in comparisons for each group, an independent-samples *t* test was applied for comparisons between groups, and ANCOVA was used to compare changes. To control for potential confounding, baseline supraspinatus tendon thickness and dominant-arm asymmetry were included as covariates in the ANCOVA model. There were no missing data in this study. All additional analyses, including subgroup and sensitivity analyses, were pre-specified in the study protocol.

### 2.5. Ethics

After being verbally informed of the details of this study, the participants received and signed a consent form. This study was conducted in accordance with the principles of the Declaration of Helsinki. The study protocol was approved by the Ethics Committee of Near East University (Approval No: YDU/2023/115-175) and was retrospectively registered at ClinicalTrials.gov (Identifier: NCT07228455). The retrospective registration was due to misunderstanding with regard to quasi-randomization

## 3. Results

Of the 46 participants who voluntarily participated in this study, 4 were excluded because they did not meet the inclusion criteria, and 2 dropped out because they could not complete the treatment. There were no statistically significant differences between the study and control groups in terms of gender, age, body mass index, secondary disease, heavy occupation, smoking, or alcohol use (*p* > 0.05). A statistically significant difference was found between the groups in terms of dominant arm (*p* < 0.05), with the right arm being favored in the study group (Table 1).

There were no statistically significant differences in demographics such as age, gender, or working conditions among the individuals who participated in our study (*p* > 0.05). Acromion morphology was predominantly Type II. In addition, there were no differences in terms of provocative/clinical tests and measurements across the two groups before the treatment. However, right arm dominance was more frequent in the intervention group. Overall, the groups were largely homogeneous, indicating that our results are unaffected by group awareness.

Table 2 shows the distributions of the provocative clinical test measurements obtained for the participants in the study and control groups and the results of the comparison between the groups.

In the study group, the results for the Neer, Hawkins, painful arc, Jobe, full-can, arm drop, and Yegarson tests were 100%, 95%, 100%, 45%, 45%, 5% positive, and 15% positive, respectively.

In the control group, the results for the Neer, Hawkins, painful arch, Jobe and full-can tests were 90%, 100%, 100%, 25%, and 20% positive, respectively. The results of the arm drop and Yergason tests were 100% negative for all patients.

There were no statistically significant differences between the groups in terms of the Neer, Hawkins, Jobe, full-can, arm drop, or Yergason tests (*p* > 0.05). No comparison could be made for the painful arch test since all the participants tested positive in both groups.

Table 3 shows the results regarding the baseline and final USG measurements for both the study and control groups.

In the study group, the acromio–humeral distance measurement was made from the most lateral tip of the acromial head to the head of the humerus. As shown in Table 3, the mean measurement of the acromio–humeral distance changed from 7.92 mm to 10.54 mm in the study group. In the control group, the AHD changed from 7.72 mm to 8.41 mm. The differences between the baseline and final values of both groups were statistically significant (*p*: 0.000). Again, the difference between the change in the baseline and final values of both groups was statistically significant (*p*: 0.000). The thickness of the supraspinatus tendon (SSp) changed from 6.48 ± 1.18 mm to 5.42 ± 1.19 mm in the study group and from 5.76 ± 0.88 mm to 5.48 ± 0.86 mm in the control group. In the study group, the thicknesses of the infraspinatus tendon (Isp), subscapularis tendon (SSc), and long biceps tendon changed from 5.09 ± 0.82 mm to 4.49 ± 0.82 mm, 4.97 ± 0.84 mm to 4.40 ± 0.87 mm, and 2.16 ± 0.45 mm to 1.77 ± 0.36 mm, respectively. In the control group, however, the thicknesses of the infraspinatus tendon, subscapularis tendon, and long biceps brachii tendon changed from 5.22 ± 0.91 mm to 4.85 ± 0.90 mm, 4.56 ± 0.74 mm to 4.14 ± 0.69 mm, and 2.01 ± 0.37 mm to 1.73 ± 0.29 mm, respectively. The thickness of the whole RC in the axial plane for the participants in the study group changed from 5.83 ± 1.03 mm to 5.09 ± 0.67 mm. In the control group, the same measurement changed from 5.58 ± 1.26 mm to 5.24 ± 1.09 mm.

The participants in the study group had greater baseline supraspinatus tendon thickness than those in the control group. When the baseline measurements of the study and control groups were compared, a statistically significant difference was found between the groups in terms of supraspinatus tendon thickness (*p* < 0.05). There was a statistically significant difference between the groups in terms of the overall thickness of the acromio–humeral distance, infraspinatus tendon, subscapularis tendon, long biceps brachii tendon, and whole RC (*p* > 0.05). The participants in the study group had greater acromio–humeral distance measurements in the post-test relative to the control group. When final differences between groups were compared, only acromio–humeral distance measurements were statistically significant (*p* < 0.05). There were no significant differences between the groups in terms of the overall thickness measurements of the supraspinatus, infraspinatus, subscapularis, long biceps brachii tendon, and RC tendons (*p* > 0.05).

The difference between the baseline and final values of the acromio–humeral distance, supraspinatus tendon, infraspinatus tendon, subscapularis tendon, long biceps brachii tendon, and RC tendons for the study group participants was statistically significant (*p* < 0.05). While the final acromio–humeral distance measurements of the study group participants increased compared to the baseline, the thicknesses of the supraspinatus, infraspinatus, subscapularis, long biceps brachii tendon, and overall rotator manchette decreased.

A statistically significant difference was found between the baseline and final values of the acromio–humeral distance, supraspinatus tendon, infraspinatus tendon, subscapularis tendon, long biceps brachii tendon, and RC tendons for the control group participants (*p* < 0.05). While the final acromio–humeral distance measurements of the control group participants were found to have increased relative to the baseline, the measurements of the thicknesses of the supraspinatus, infraspinatus, subscapularis, long biceps brachii, and RC tendons were found to have decreased.

Upon comparing the baseline and final changes in the study and control groups, statistically significant differences were found with regard to the acromio–humeral distance, supraspinatus tendon, and overall thickness of the rotator manchette (*p* < 0.05). For the study group, the increase in acromio–humeral distance and the decrease in the general thickness of the supraspinatus tendon and other RC tendons were found to be greater relative to the control group. There were no statistically significant differences between the groups in terms of the levels of change in the infraspinatus, subscapularis, and long biceps brachii tendon measurements (*p* > 0.05).

As shown in Table 4, when the baseline and final VAS measurements were examined, the resting VAS score of the participants in the study group changed from 2.05 ± 2.09 to 0.00 ± 0.00; the night pain VAS score changed from 7.90 ± 1.48 to 1.95 ± 1.39; and the activity pain VAS scores changed from 3.45 ± 3.14 to 0.00 ± 0.00. These differences were statistically significant (*p* < 0.05). In the control group, the resting VAS score changed from 2.65 ± 2.39 to 0.40 ± 0.99; the nocturnal pain VAS score changed from 8.10 ± 1.77 to 4.15 ± 1.63; and the VAS scores of activity pain changed from 2.90 ± 2.65 to 0.85 ± 1.35. These differences were significant (*p* < 0.05). While there were no significant differences between the values for the two groups before the treatment (*p* > 0.05), a significant difference was found between the two groups in favor of the study group in terms of the final night pain and resting pain scores (*p* < 0.05). There were no statistically significant differences between the groups in terms of resting VAS measurements (*p* > 0.05).

Table 5 shows changes in the scores of the McGill Pain Questionnaire and Fear Avoidance Beliefs Questionnaire. In the study group, the McGill Pain Questionnaire score changed from 68.10 ± 14.87 to 42.05 ± 9.84, while The Fear Avoidance Beliefs Questionnaire score changed from 42.65 ± 21.81 to 12.65 ± 9.23. In the control group, the McGill Pain Questionnaire score changed from 71.85 ± 19.58 to 55.15 ± 15.40, and the Fear Avoidance Beliefs Questionnaire score changed from 55.50 ± 28.46 to 46.80 ± 28.92. While there were no statistically significant differences between the groups before the treatment, a statistically significant difference emerged after the treatment. (*p* < 0.05) Statistically significant changes in the results were found for both groups when comparing the baseline and final measurements (*p* < 0.05). This difference was more pronounced in the study group.

Table 6 reveals the baseline and final results regarding the Arm, Shoulder and Hand Problems Questionnaire (DASH) scores in both groups.

The DASH score for the study group changed from 37.20 ± 13.44 to 9.33 ± 6.12. In the control group, the DASH score changed from 44.95 ± 16.84 to 33.06 ± 15.59.

Before the treatment, there were no statistically significant differences between the groups (*p* > 0.05); however, the DASH score was higher in the control group. After treatment, the DASH score was found to be lower in the study group than in the control group (*p* < 0.05).

There was a statistically significant difference between the groups with regard to the DASH score (*p* < 0.05). Accordingly, the scores of both groups were found to be lower than the baseline.

When the changes in the DASH scores were compared, a statistically significant difference was found between the groups (*p* < 0.05). The decrease in the DASH score was found to be greater in the study group than in the control group.

### Safety Evaluation

No harmful or undesirable events were reported in either the intervention or the control group during the trial.

## 4. Discussion

SAPS accounts for approximately 44–65% of shoulder injuries [18,19]. The supraspinatus (along with the long head of the biceps tendon) and, to a lesser extent, the infraspinatus tendons pass through the space between the lower edge of the acromion and the head of the humerus as they approach the tuberculum majus of the humerus. This area, which normally allows the RC tendons to move smoothly due to scapulo-humeral rhythm and proper muscle function, can become narrowed for various reasons, causing impingement. As a result, micro-traumas, to which tendons are easily exposed, lead to deterioration of the local environment, resulting in inflammation, degeneration, thickening, and tears for various reasons. Continuous or use-related pain then results in detriments in everyday activities and reduces quality of life. Our study was designed to test the hypothesis that a combined exercise program, including strengthening of the shoulder depressors, scapular stabilization, and proprioceptive training, could change AHD and its symptoms. Therefore, we aimed to reduce the impact of impingement syndrome through fixing scapula–humeral rhythm disturbance, a significant public health concern. The improvements observed may be the result of the combined effect of all the components of the program rather than a single exercise.

AHD measured via X-ray is generally reported to be around 10 mm (7–14 mm), When measured using MRI, this value appears to be about 1.2 mm narrower than that obtained via plain radiography [20]. Sripathi et al. associate a distance less than 6 mm with a full-thickness RC tear [21]. The values obtained using USG vary; they are typically reported to be 9–12 mm in healthy individuals, while in different tendon pathologies, they range between 6 and 10 mm [22]. Yuan X et al. found an AHD of 1.07 cm+/−0.21 to 1.12 cm+/−0.24 at a neutral distance [8]. Based on the USG measurements obtained before and after treatment in this study, there was a statistically significant increase in AHDs in both the study and control groups. The AHD increased from 7.92 mm to 10.54 mm in the study group and from 7.72 mm to 8.41 mm in the control group, with the study group exhibiting a greater increase. Studies on the relationship between AHD and SAPS have yielded conflicting results. Some authors report that AHD is not related to symptoms, while others claim that it is. Navarro-Ledesma et al. found no relationship between AHD at 0 and 60 degrees of abduction and pain or disability [19]. Others, such as Savoie et al. [18], have reported significant improvements in AHD, shoulder function, and pain. Conversely, Park et al., in their meta-analysis, found no relationship between AHD and pain in people with SAPS. [23] Similarly, Dede et al. found no differences in AHD between healthy individuals and patients with partial tears, whereas the difference between healthy individuals and those with total tears was significant [24]. Interestingly, Hunter et al. reported greater values for both AHD and SSp thickness in symptomatic patients relative to the values found for the healthy ones, similar to what was observed in our study [25]. The contradictory findings in the literature may stem from differences in the measurement methods, study populations, or treatment modalities used. An easy way to understand this is through studies where AHD has been expanded via arthroscopic decompression surgery (SAD). The results remain inconclusive; some studies report positive outcomes, while others indicate the opposite. Ketola et al. found that the long-term effectiveness of SAD surgery was not superior to that of conservative approaches over a 5-year follow-up [26]. In contrast, in their 6-month follow-up, Jacobsen et al. reported better results for patients who underwent SAD according to national guidelines [27]. This study reveals positive changes in both groups, with more pronounced results in the study group. Critically, not all the pain related to the RC is caused by impingement; other connective-tissue elements, including the subacromial bursa, are also involved. Therefore, expecting full results from only debriding the upper bony contour may not be entirely reasonable.

Although the relationship between RC thickness and AHD has been studied in the literature [25], we have not found any studies ultrasonographically investigating whether this change is affected by treatment. In fact, the exercise approaches are mostly applied for SAPS, in which case intensive training has usually been performed for the RC muscles. However, there is limited information about the effectiveness of exercise training in depressing the humeral head. In their 2010 study, Tate et al. had individuals with shoulder impingement syndrome undergo exercise training for the rotator cuff muscles. Ultimately, they argued that the training was an effective method of reducing the symptoms [28]. Boudreau et al., in their EMG-oriented study, reported that strengthening the glenohumeral adductor muscles, along with the RC muscles, is an effective method for ameliorating the existing pathology by improving the efficiency of the RC muscles. But they did not observe any statistical differences regarding pain, function, or USG measurement. Though mentioned, the method and place of measurement were not given in detail [29]. The authors of another study reported that humeral head depressor training, which focuses only on the pectoral and latissimus dorsi muscles, is an effective method for the short-term improvement of the shoulder joint [30]. These studies did not assess the long-head biceps muscle or serratus muscles, which are important humeral-head-depressing muscles. Similarly, Çelik et al. found a relationship between pain and weaknesses in the supraspinatus and deltoid muscles [31]. Considering the findings of our study, both RC and humeral head depressor training result in decreased RC tendon thicknesses and help ameliorate AHD caused by impaired biomechanics. While both groups showed a decrease in tendon thickness and an increase in AHD, the difference in the study group that underwent a combined specific program to depress the humeral head was more prominent. This may be compatible with the efficacy of the applied strategy of managing thickening and easing RC mobility in the study group. Studies examining the correlation between thickness and AHD have reported variable results. Two studies demonstrated a relationship in their USG analyses while trying to determine the distance of AHD and its relationship with the thickness of SSp in SAPS patients [25,32]. Contrary to other studies, Ishigaki et al. did not find a relationship between AHD and SSp tendon thickness, a result that is consistent with our study [33]. Similarly, Dede et al. could not find any differences regarding tendon thickness between patients and controls [24]. Another study conducted on patients with non-traumatic shoulder pain found no relationship between thickness and symptoms [34].

Pain that wakes one up from sleep is common in patients with SAPS, and it is also intense, especially during the day, as confirmed in our study group. This pain may be related to inflammation and overhead activities engaged in during the day. Therefore, it may cause sleep problems, depression, and anxiety. The data obtained in this study reveal that pain levels during rest, activity, and nighttime were significantly reduced in both groups, with a greater difference for the study group. We believe that this phenomenon is related to the improvement of the deteriorated shoulder biomechanics in these individuals and is a result of an increased AHD, which may result in the elimination of existing mechanical stress. Furthermore, the training program employed may influence AHD and RC tendons, both structurally and functionally. The scores on the McGill Pain Questionnaire, a multidimensional assessment tool that provides more information about the characteristics of the pain felt, decreased significantly in the study group, which was in accordance with the VAS pain score [10]. This finding suggests that the applied treatment method has a positive effect not only on pain intensity but also on pain perception among individuals with SAPS. Accordingly, the VAS and McGill Pain Questionnaire scores show that the study group participants may have reduced their pain sensation bio-psychosocially. The results obtained in other studies vary. Eraslan et al. reported that exercise training for the subscapular and glenohumeral muscles, undertaken by individuals with SAPS, was an effective method of both AHD improvement and pain reduction, a finding compatible with this study [35]. In contrast, according to a meta-analysis by Park et al., one study found no relationship between AHD and pain, while the other two studies reported a weak relationship, indicating less pain and disability following an increase in AHD [23]. In terms of resting pain, there was no statistical difference between the groups, although there was a greater decrease in the study group. The same study reported that SAPS patients experience more pain during the day due to excess overhead activity [36]. However, it should be kept in mind that not all the studies used the same protocols.

Upon reviewing the literature, our attention was drawn to the presence of upper-extremity dysfunction and disability in SAPS, with reports showing that treatment led to improvements in disability and functionality [37,38]. One study revealed that increases in the level of rest- and activity-related pain exacerbate both the limitations in daily activities and the level of fear of motion. According to the DASH results obtained in our study, there is a significant relationship between pain levels and functional limitations and kinesiophobia. Kinesiophobia in individuals with SAPS has been reported previously [39]. In this study, kinesiophobia was present in both groups from the outset; later, there was a significant decrease in both groups. The study group participants were indeed found to exhibit a greater reduction. Araco et al. reported that activity avoidance behavior was more common in individuals with pain and disability symptoms who suffered from SAPS [40]. Additionally, Karaartı et al. drew attention to the kinesiophobia that develops due to pain and argued that the existing pain poses an obstacle to recovery by provoking activity avoidance behavior [41]. Accordingly, we believe that the decrease in activity-related pain levels in the study group enabled the individuals to move away from activity avoidance behavior. This may have been affected by the increased self-confidence of the individuals during the activity. Thus, pain is not only a symptom of shoulder problems but also an essential factor that triggers behavioral avoidance. Our findings are consistent with the literature [37,39,42].

This study shows that the study group participants experienced greater improvement compared to the control group participants in terms of upper-extremity functions and disability. The results suggest that the combined exercise program, including humeral head depressor strengthening, scapular stabilization, and proprioceptive training, may provide an effective strategy for managing inflammation and dysfunction in the RC, particularly the supraspinatus tendon, which could be affected by narrowed AHD and mechanical stress. This treatment may help reduce mechanical stress and improve shoulder biomechanics, potentially allowing the muscles and tendinous structures to adapt and strengthen. Overall, these improvements may contribute to ameliorating disability and enhancing pain relief and functional outcomes.

## 5. Limitations

A significant limitation of this study is that a long-term follow-up of the participants could not be conducted. This may be an important obstacle to the extensibility of the results

Another limitation was that this study was conducted at a single center, and the number of allocated patients was limited.

## 6. Conclusions

In our study, the exercise program targeting humeral head depressor muscle training applied in the intervention group resulted in increased acromio-humeral distance and reduced supraspinatus tendon thickness, which we associate with the eased mobilization of the RC, improved scapulo-humeral rhythm, and decreased pain and functional disability in individuals with subacromial pain syndrome. In addition, this program was found to have positive effects on kinesiophobia. These findings suggest that, in addition to a classical rehabilitation program, a specific exercise program designed to increase the strength and contractility of humeral head depressor muscles may have an important impact on SAPS rehabilitation. However, this study has certain methodological limitations, including a relatively small sample size and a lack of long-term follow-up assessments, which should be considered when interpreting the results.

## Figures and Tables

**Table 1:** Comparison of participants’ socio-demographic characteristics.

	Intervention		Control		X	*p*
	*n*	%	*n*	%		
Gender						
Female	11	55.0	11	55.0	0.000	1.000
Male	9	45.0	9	45.0		
Age	40.45 ± 8.38		41.60 ± 13.26		−0.328	0.745
BMI						
Normal	6	30.0	10	50.0	2.067	0.356
Slightly overweight	8	40.0	7	35.0		
Obese	6	30.0	3	15.0		
Active arm						
Right	18	90.0	10	50.0	7.619	0.006*
Left	2	10.0	10	50.0		
Secondary disease						
Yes	2	10.0	3	15.0	0.229	0.633
No	18	90.0	17	85.0		
Laborious occupation						
Yes	3	15.0	4	20.0	0.173	0.677
No	17	85.0	16	80.0		
Smoker						
Yes	8	40.0	5	25.0	1.026	0.311
No	12	60.0	15	75.0		
Alcohol user						
Yes	3	15.0	2	10.0	0.229	0.633
No	17	85.0	18	90.0		

**Table 2:** Comparison of participants’ provocative clinical test measurements.

		**Intervention** **(*n* = 20)**		**Control** **(*n* = 20)**		**X^2^**	***p***
		***n***	**%**	***n***	**%**		
Neer test	Positive	20	100.0	18	90.0	2.105	0.147
	Negative	0	0.0	2	10.0		
Hawkins test	Positive	19	95.0	20	100.0	1.026	0.311
	Negative	1	5.0	0	0.0		
Painful arch	Positive	20	100.0	20	100.0	-	-
Jobe test	Positive	9	45.0	5	25.0	1.758	0.185
	Negative	11	55.0	15	75.0		
Full-can test	Positive	9	45.0	4	20.0	2.849	0.091
	Negative	11	55.0	16	80.0		
Arm drop test	Positive	1	5.0	0	0.0	1.026	0.311
	Negative	19	95.0	20	100.0		
Yergason test	Positive	3	15.0	0	0.0	3.243	0.072
	Negative	17	85.0	20	100.0		

**Table 3:** Comparison of participants’ pre- and post-treatment USG measurements.

	Group	Pre-Treatment			Post-Treatment			*p*_3_	F	*p*_4_	η^2^
		$\bar{x}$	s	*p*_1_	$\bar{x}$	s	*p*_2_				
Acromion tip– humeral head	Intervention	7.92	0.87	0.518	10.54	1.53	0.000 *	0.000 *	53.030	0.000 *	0.589
	Control	7.72	1.10		8.41	1.00		0.000 *			
M. Supraspinatus tendon	Intervention	6.48	1.18	0.036 *	5.42	1.19	0.856	0.000 *	16.824	0.000 *	0.313
	Control	5.76	0.88		5.48	0.86		0.000 *			
M. İnfraspinatus tendon	Intervention	5.09	0.82	0.651	4.49	0.82	0.194	0.000 *	3.699	0.062	0.091
	Control	5.22	0.91		4.85	0.90		0.001 *			
M. Subscapularis tendon	Intervention	4.97	0.84	0.109	4.40	0.87	0.301	0.000 *	1.390	0.246	0.036
	Control	4.56	0.74		4.14	0.69		0.000 *			
M. Biceps brachii Long-Head tendon	Intervention	2.16	0.45	0.271	1.77	0.36	0.665	0.000 *	0.297	0.589	0.008
	Control	2.01	0.37		1.73	0.29		0.000 *			
Rotator cuff group Tendons’ general thicknesses	Intervention	5.83	1.03	0.487	5.09	0.67	0.604	0.000 *	4.808	0.035 *	0.115
	Control	5.58	1.26		5.24	1.09		0.004 *			

* *p* < 0.05. *p*_1_: Comparison of pre-test scores between groups (independent-samples *t*-test). *p*_2_: Comparison of post-test scores between groups (independent-samples *t*-test). *p*_3_: Comparison of pre-test–post-test scores within groups (paired-sample *t*-test). *p*_4_: Comparison of changes in pre-test–post-test scores in both groups (ANCOVA).

**Table 4:** Comparison of participants’ VAS measurements before and after treatment.

	Group	Pre-Treatment			Post-Treatment			*p*_3_	F	*p*_4_	η^2^
		$\bar{x}$	s	*p*_1_	$\bar{x}$	s	*p*_2_				
VAS Rest	Intervention	2.05	2.09	0.403	0.00	0.00	0.080	0.000 *	2.476	0.124	0.063
	Control	2.65	2.39		0.40	0.99		0.000 *			
VAS Night pain	Intervention	7.90	1.48	0.701	1.95	1.39	0.000 *	0.000 *	26.877	0.000 *	0.311
	Control	8.10	1.77		4.15	1.63		0.000 *			
VAS Activity pain	Intervention	3.45	3.14	0.553	0.00	0.00	0.008 *	0.000 *	10.823	0.002 *	0.226
	Control	2.90	2.65		0.85	1.35		0.000 *			

* *p* < 0.05. *p*_1_: Comparison of pre-test scores between groups (independent-samples *t*-test). *p*_2_: Comparison of post-test scores between groups (independent-samples *t*-test). *p*_3_: Comparison of pre-test–post-test scores within groups (paired-sample *t*-test). *p*_4_: Comparison of changes in pre-test–post-test scores in both groups (ANCOVA).

**Table 5:** Comparison of participants’ pre- and post-treatment McGill Pain Questionnaire and Fear Avoidance Beliefs Questionnaire measurements.

	Group	Pre-Treatment			Post-Treatment			*p*_3_	F	*p*_4_	η^2^
		$\bar{x}$	s	*p*_1_	$\bar{x}$	s	*p*_2_				
McGill Pain Questionnaire	Intervention	68.10	14.87	0.499	42.05	9.84	0.003	0.000 *	15.383	0.000 *	0.294
	Control	71.85	19.58		55.15	15.40		0.000 *			
Fear Avoidance Beliefs Questionnaire	Intervention	42.65	21.81	0.117	12.65	9.23	0.000 *	0.000 *	32.911	0.000 *	0.471
	Control	55.50	28.46		46.80	28.92		0.006 *			

* *p* < 0.05. *p*_1_: Comparison of pre-test scores between groups (independent-samples *t*-test). *p*_2_: Comparison of post-test scores between groups (independent-samples *t*-test). *p*_3_: Comparison of pre-test–post-test scores within groups (paired-sample *t*-test). *p*_4_: Comparison of changes in pre-test–post-test scores in both groups (ANCOVA).

**Table 6:** Comparison of participants’ pre- and post-treatment Arm, Shoulder, and Hand Problems Questionnaire (DASH) measurements.

	Group	Pre-Treatment			Post-Treatment			*p*_3_	F	*p*_4_	η^2^
		$\bar{x}$	s	*p*_1_	$\bar{x}$	s	*p*_2_				
DASH	Intervention	37.20	13.44	0.116	9.33	6.12	0.000 *	0.000 *	113.912	0.000 *	0.755
	Control	44.95	16.84		33.06	15.59		0.000 *			

* *p* < 0.05. *p*_1_: Comparison of pre-test scores between groups (independent-samples *t*-test). *p*_2_: Comparison of post-test scores between groups (independent-samples *t*-test). *p*_3_: Comparison of pre-test–post-test scores within groups (paired-sample *t*-test). *p*_4_: Comparison of changes in pre-test–post-test scores in both groups (ANCOVA).

## Data Availability

Data are unavailable because of legal/privacy restrictions. The trial protocol and statistical analysis plan are not publicly available but can be provided by the corresponding author upon reasonable request.

## Abbreviations

The following abbreviations are used in this manuscript:

SAPS	Subacromial Pain Syndrome
RC	Rotator Cuff
SB	Subacromial Bursa
AHD	Acromio-Humeral Distance
MRI	Magnetic Resonance Imaging
USG	Ultrasonography
VAS	Visual Analog Scale
SPSS	Statistical Package for the Social Sciences
SAD	Arthroscopic Decompression Surgery
